# Utilization of Replication-Competent XMRV Reporter-Viruses Reveals Severe Viral Restriction in Primary Human Cells

**DOI:** 10.1371/journal.pone.0074427

**Published:** 2013-09-13

**Authors:** Christina Martina Stürzel, David Palesch, Mohammad Khalid, Silke Wissing, Nicole Fischer, Jan Münch

**Affiliations:** 1 Institute of Molecular Virology, Ulm University Medical Centre, Ulm, Germany; 2 Gladstone Institute of Virology and Immunology, University of California San Francisco, San Francisco, California, United States of America; 3 Institute for Medical Microbiology, Virology and Hygiene, University Medical Center Hamburg-Eppendorf, Hamburg, Germany; Institut Pasteur Korea, Korea, Republic Of

## Abstract

The gammaretrovirus termed xenotropic murine leukemia virus-related virus (XMRV) was described to be isolated from prostate cancer tissue biopsies and from blood of patients suffering from chronic fatigue syndrome. However, many studies failed to detect XMRV and to verify these disease associations. Data suggesting the contamination of specimens in particular by PCR-based methods and recent reports demonstrating XMRV generation via recombination of two murine leukemia virus precursors raised serious doubts about XMRV being a genuine human pathogen. To elucidate cell tropism of XMRV, we generated replication competent XMRV reporter viruses encoding a green fluorescent protein or a secretable luciferase as tools to analyze virus infection of human cell lines or primary human cells. Transfection of proviral DNAs into LNCaP prostate cancer cells resulted in readily detectably reporter gene expression and production of progeny virus. Inoculation of known XMRV susceptible target cells revealed that these virions were infectious and expressed the reporter gene, allowing for a fast and highly sensitive quantification of XMRV infection. Both reporter viruses were capable of establishing a spreading infection in LNCaP and Raji B cells and could be easily passaged. However, after inoculation of primary human blood cells such as CD4 T cells, macrophages or dendritic cells, infection rates were very low, and a spreading infection was never established. In line with these results we found that supernatants derived from these XMRV infected primary cell types did not contain infectious virus. Thus, although XMRV efficiently replicated in some human cell lines, all tested primary cells were largely refractory to XMRV infection and did not support viral spread. Our results provide further evidence that XMRV is not a human pathogen.

## Introduction

In 2006 the first human gammaretrovirus within the retroviridae family was reported to be identified in human prostate cancer tissue. Because of its sequence homology to murine leukemia viruses this novel virus was termed xenotropic murine leukemia virus-related virus (XMRV) [1]. Later, XMRV DNA was also presumably detected in blood samples from patients diagnosed with chronic fatigue syndrome (CFS) [2]. It has then been shown that mouse DNA present in trace amounts in various PCR kits resulted in false positive XMRV PCR reactions [3,4]. Since then, published studies addressing XMRV prevalence applying well-controlled PCR protocols to prostate cancer [5,6], CFS [7] or diagnostic samples from other disorders like e.g. autism [8] failed to detect XMRV (reviewed here [9–12]). In support of these findings, it was recently demonstrated that the virus originated when two mouse leukemia viruses underwent recombination during experimental passage of a human prostate tumor xenograft in mice [13]. Furthermore, CFS samples previously reported to contain XMRV DNA were reexamined and no XMRV was detected [7]. In summary, subsequent studies cast massive doubts on the association of XMRV with CFS or prostate cancer [14–18] but XMRV remains a replication competent virus with novel biological features.

To be a genuine human pathogen, XMRV must be capable of infecting and replicating in human cells. Although XMRV tropism and replication has extensively been studied in several cell lines of human origin [19–21], our knowledge about XMRV infection and spread in primary human cells or tissues is quite limited. XMRV infects and replicates efficiently in prostate cancer cell lines such as LNCaP, DU145 and PC-3 cells [22,23], in human embryonic kidney (HEK) 293 cells [20], HeLa cervical cancer cells [21], Jurkat T cells [20], or the B cell line BJAB [19]. Only three articles to date addressed the replicative capacity of XMRV in human peripheral blood mononuclear cells (PBMC) [24–26]. These studies demonstrated that activated PBMC could be infected with XMRV derived from chronically infected 22Rv1 cells [24,25], or LNCaP cells transfected with proviral DNA [26] but only when very high viral doses were used for inoculation. In addition, Curriu and colleagues recently showed that human lymphoid tissue can be infected when extreme high viral incoula were applied [27]. In all studies no or only low amounts of infectious progeny virions were released into the supernatant indicating potent restriction of XMRV replication in PBMC and human lymphoid tissue cells [24–26]. The XMRV replication block in PBMC has been attributed to restriction factors APOBEC 3G and 3F [25,28], two cellular cytidine deaminases that induce inactivating hyper-mutations in the genomes of retroviruses and retrotransposons [29,30]. Another restriction factor that blocks XMRV release is tetherin/BST-2/CD317 that tethers budding virions to the cell surface [31]. However, unlike HIV-1 that encodes Vif and Vpu proteins to counteract the antiviral activity of APOBEC3 or tetherin [32,33], XMRV has not evolved means to neutralize these two cellular restriction factors. This is in contrast to other infectious murine leukemia viruses that utilize a glycosylated Gag protein to antagonize APOBEC3 [34]. In line with these findings, introducing the glycosylated Gag of moloney murine leukemia virus into XMRV facilitates XMRV replication [35].

In this study, we analyzed XMRV tropism and replication in human cell lines and primary cells using novel replication-competent XMRV reporter viruses. These viruses express the green fluorescence protein (GFP) or a secretable luciferase derived from the copepod *Gaussia* *princeps* (GLUC) downstream of the envelope reading frame. Our results obtained with XMRV-GFP and -GLUC confirm previous data demonstrating efficient XMRV replication in various human cell lines. However, contrary to established cell lines, XMRV is severely restricted and does not efficiently replicate in a variety of primary human blood cells including CD4, CD8, dendritic cells or macrophages. Our findings are in good concordance with previous studies and argue against XMRV as a genuine human pathogen since it does not spread in primary human cells.

## Material and Methods

### Generation of XMRV reporter viruses

The pcDNA3.1 XMRV isolate VP62 was kindly provided by Robert Silverman. XMRV-Δ5U3-VP62 was first cloned into the pCR2.1-TOPO vector using unique NotI and HindIII sites. Next, 5’ and 3’ LTRs were completed by cloning spliced overlap extension PCR derived DNAs. First, the U3 region from the 3’ LTR was amplified using primers pNotI-U3 (5’-GCGGCCGCTGAAAGACCCCACCATAAGGCT-3’) and p3Rep (5’-GTCTATCGGATGACTGGCGCG-3’). The repeat and the U5 region from the 5’ LTR were amplified using primers p5Rep (5’-CGCGCCAGTCATCCGATAGAC) and pSacII (5’-CGTCAGTTCTTCCGCGGAACCGCCAGAT-3’). The left- and right-half PCR products were gel purified, mixed in equimolar amounts, and subjected to a second PCR with primers pNotI-U3 and pSacII. To generate the complete 5’-LTR in the full-length XMRV-VP62 genome, the PCR product was cloned into the pCR2.1-TOPO-XMRV-VP62 construct by using the unique NotI and SacII sites.

An *IRES-GFP* cassette was PCR amplified from pIRES2-eGFP (Clonetech) using primers env-IRES-GFP sense 5’-gtgaataaATTCTGCAGTCGACGGTAC-3’ and IRES-GFP reverse 5’-atagcatcacgtgaaTTACTTGTACAGCTCGTCCAT-3’. The PCR product was cloned into the pcDNA3.1-XMRV-VP62 opened by PmlI at position 7691. Next, the env-IRES-GFP_3’LTR fragment was cloned by using BstBI and HindIII restriction sites into pCR2.1-TOPO-XMRV-VP62, resulting in pCR2.1-TOPO-XMRV-VP62-GFP.

To generate proviral XMRV-VP62-GLUC reporter constructs, the IRES region was PCR amplified using the primers 5’-BstBI (GACAAAAATTGTTCGAATCAGGACA) and 3’ IRES-GLUC (GACTCCCATGGTTGTGGCCATATTA). The *GLUC* gene was PCR amplified with primers 5’ GLUC (GCCACAACCATGGGAGTCAAAGTTCTGTT) and 3’-BsrGI (AAATCTTTTAGTCACCACCGGCCCCCTTGAT). Both PCR products were gel purified, mixed in equimolar amounts, and subjected to a second PCR with primers 5’-BstBI and 3’-BsrGI. The obtained *env-IRES-GLUC* cassette was cloned into pCR2.1-TOPO-XMRV-VP62-GFP resulting in the generation of pCR2.1-TOPO-XMRV-VP62-GLUC.

The XMRV-VP62-env*-IRES-GFP construct was generated using the Quik Change II XL Site Directed Mutagenesis Kit (Agilent Technologies) and the primers 5’-del76 GATAATTATGGGGATCTGGTGAGGGCAGGAGC and 3’-del76 GCTCCTGCCCTCACCAGATCCCCATAATTATC. The construct contains a thymine deletion at position 76 which results in a frameshift in the *env*-encoding region and the introduction of a stop codon at triplet position 25. All constructs were sequenced on both strands and complete plasmid sequences for all XMRV proviral plasmids described herein are available on request.

### Cell Culture

Cell lines were obtained from ATCC if not indicated otherwise. A3.01 and PM1 cell lines were obtained from the National Institutes of Health AIDS Research and Reference Reagent Program (Germantown, MD, USA). Jurkat, JTAg, CEM, SupT1, BJAB, Raji, Jurkat, and DU145 cells were cultured in RPMI medium (10% heat-inactivated FCS, 120 µg/ml Penicillin, 120 µg/ml Streptomycin, 2 mM L-Glutamine). LNCaP cells and LNCaP DERSE-iGFP (kindly provided by Vineet N. KewalRamani, National Cancer Institute, Frederick, USA) [27] were cultured in RPMI medium (10% heat-inactivated FBS, 120 µg/ml Penicillin, 120 µg/ml Streptomycin, 2 mM L-Glutamine, 10 mM HEPES, 1 mM Sodium Pyruvate, 4.5 g/l D-Glucose, 1.5 g/l Sodiumhydrogencarbonate). HEK-293T, HeLa, U87 and U373 were cultured in DMEM medium (10% heat-inactivated FCS, 120 µg/ml Penicillin, 100 µg/ml Streptomycin, 2 mM L-Glutamine). Studies involving primary human cells were reviewed and approved by the University of Ulm Institutional Review Board, and individuals and/or their legal guardians provided written informed consent prior to donating blood. PBMCs were isolated by ficoll-density centrifugation. T cells were stimulated using 1 µg/ml PHA (Oxoid, Basingstoke, UK) and 10 ng/ml IL-2 (Miltenyi Biotech, Bergisch-Gladbach, Germany) for three days. Monocytes were selected by adherence and cultured for six days with 100 ng/ml GM-CSF to obtain M1-macrophages, 100 ng/ml M-CSF for M2-macrophages, and 100 ng/ml GM-CSF and 25 ng/ml IL-4 for monocyte-derived dendritic cells (all purchased from R&D Systems, Minneapolis, MN, USA). CD19^+^ primary B cells were MACS-separated (Miltenyi Biotech, Bergisch-Gladbach, Germany) and stimulated for three days using 200 U/ml IL-4, 200 ng/ml IL-10, and 0.5 µg/ml CD40L (all obtained from PROSPEC, East Brunswick, NJ, USA). PBMC-derived cells were cultivated in RPMI complete medium.

### Generation of virus stocks

LNCaP cells were seeded into 24-well plates. At 50-60% confluency, cells were transfected with 2 µg of proviral XMRV DNA per well using Lipofectamine 2000 reagent according to the manufacturer’s instructions (Invitrogen). Supernatants containing XMRV particles were collected between 17 and 24 days post transfection, filtered, aliquoted and stored at -80°C. Viral stocks were then titrated on LNCaP and stocks with the highest titer identified.

### Reverse transcriptase (RT) assay

The C-type RT assay activity kit (CAVIDI) was used according to manufacturer’s protocol to measure RT activity in cell culture supernatant.

### Quantification of XMRV titers by real-time PCR

DERSE-iGFP cells were exposed to filtered culture supernatant from transfected LNCaP cells. 500 µl of supernatant was added to 5x10^4^ DERSE-iGFP cells which were scored for XMRV viral titers seven days post infection by real-time PCR analysis. Briefly, RNA of 500 µl supernatant or virus stock were isolated with RNAbee reagents (AMS Biotechnology) according to manufacture instructions. RNA was resuspended in 50 µl H_2_O. 9 µl RNA was used for cDNA synthesis using Superscript III enzyme (Invitrogen). cDNA levels were quantified using a Qiagen Rotorgene Q5plex instrument and Rotorgene 1.7 software. Reactions were performed in microtubes containing 5 µl 2x SyBr Green mastermix (Fermentas), 3.8 µl H_2_O, 0.1 µl primer (100 pmol/µl) and 1 µl cDNA (1:5 diluted). XMRV specific primers have been published earlier [21]. PCR efficiency of the primer set (0.98) was determined based on standard curves of serial 10 fold dilutions of DNA isolated from 5x10^5^ 22Rv1 cells. Ct values (determined by using the Rotorgene Software version 1.7) were plotted against the log_10_ value of template concentration.

### Analysis of XMRV-GLUC transfected and infected cells

Indicated cells were inoculated with serial dilutions of XMRV-GLUC derived from provirally transfected LNCaP. After 16 hours, cells were pelleted, washed (to remove inoculum GLUC activity), and supplemented with fresh medium. Supernatants (50 µl) were taken immediately after the first washing step and at regular intervals thereafter, and stored at -20°C. Supernatants of XMRV-GLUC transfected cells were obtained as described above. GLUC activities in all supernatants were determined using the *Gaussia* -Juice Kit (P.J.K.) as recommended by the manufacturer. Reporter enzyme activities were measured using the Orion microplate luminometer (Berthold). GLUC activities obtained in the washing control were subtracted from GLUC activities of supernatants collected thereafter.

### Analysis of XMRV-GFP transfected and infected cells

10^5^ LNCaP, HEK-293T, HeLa, U87, U373, DU-145, Jurkat, Raji, BJAB, PM1, SupT1, A3.01, CEM and Jtag20 cells were seeded into 24-well plates in 500 µl of growth media. Cells were infected with 100 µl of 17^th^ dpt XMRV-GFP virus stock and three days later cells were fixed in PFA and subjected to flow cytometry for GFP expression using a FACS Canto II cytometer (BD). Transfected cells were handled the same way.

### Effect of AZT on XMRV infection

2 x 10^4^ Raji cells were seeded into 96-well plates in 90 µl media. AZT was added in a volume of 10 µl to achieve the final concentration of 0.7, 2.1, 6.2, 19, 56, 167 and 500 nM. Subsequently, cells were incubated for 1 hour and infected with 10 µl of XMRV-GLUC virus. After overnight incubation cells were washed to remove inoculum GLUC activity. Cellular supernatants were monitored for GLUC activity at indicated time points. IC50 was determined with PRISM software.

### XMRV spreading infection

A total of 10^5^ Raji cells in 500 µl medium were seeded into 24-well plates and infected with serial dilutions of XMRV-GFP virus. Every 24 hrs, GFP expression was analyzed by flow cytometry.

### Plasma membrane labeling

A total of 10^5^ Raji cells, in 500 µl of the media, were seeded into 24-well plates and infected with 100 µl of 5 fold diluted 17^th^ dpt XMRV-GFP virus. Two days post infection the cells were washed with PBS and stained with Cell Mask orange according to the manufacturer’s instructions (Invitrogen). Thereafter the cells were fixed with 4% PFA, resuspended in 50 µl PBS and transferred in an IBIDI chamber slide (µ-slide 8 well; IBIDI). The samples were mounted with Vectashield DAPI and subjected to fluorescence microscopy using a LSM 710 laser scanning microscope (Zeiss).

### Statistics

Data were analyzed using two-tailed Student’s t test (PRISM package version 5.0 (Abacus Concepts, Berkeley, CA)).

## Results and Discussion

**Figure 1:**
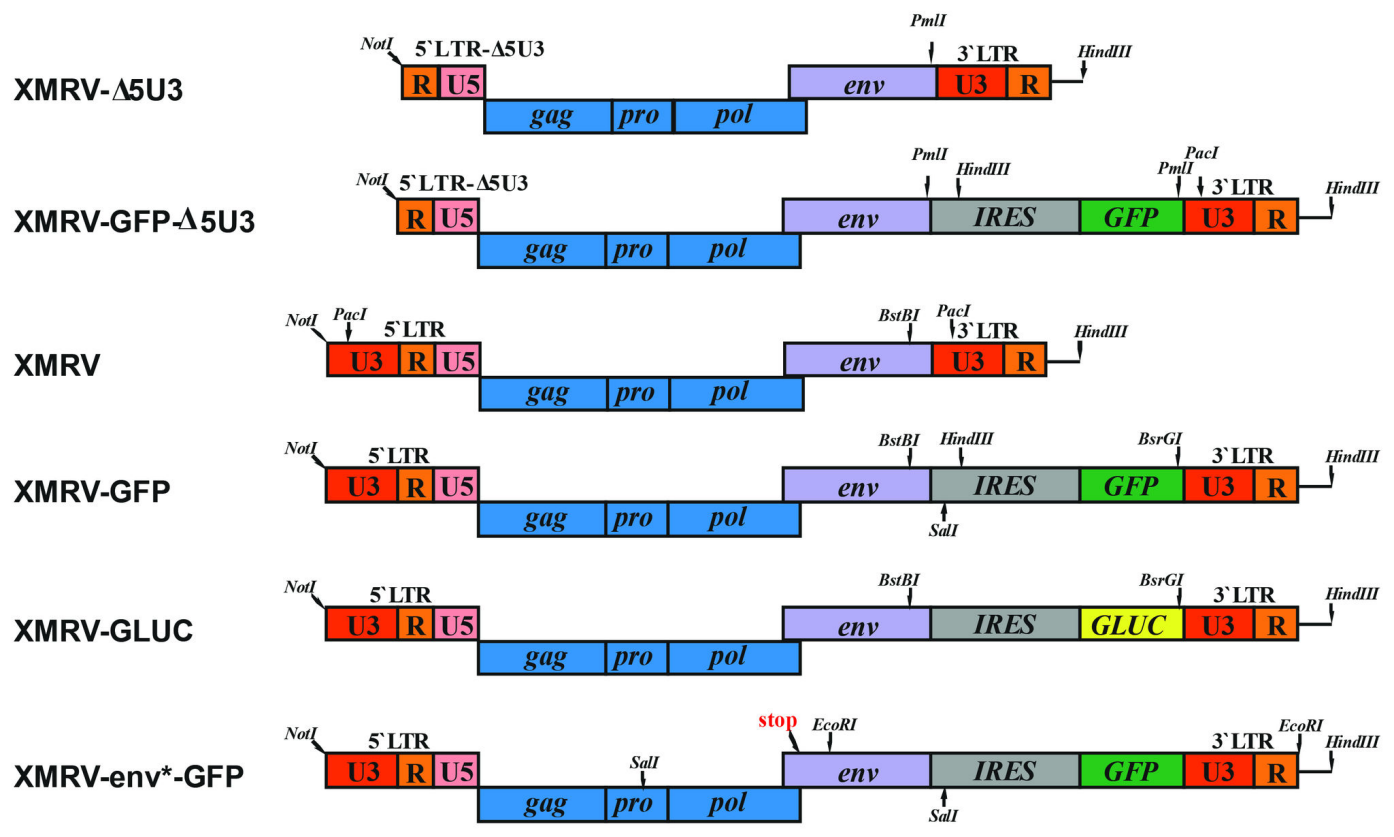
Overview of proviral XMRV reporter viruses. Shown are the genomic organizations of the parenteral XMRV provirus (XMRV-∆5U3), the provirus with the reconstituted 5`LTR (XMRV), and the reporter viruses coexpressing env and *GFP* or *GLUC* via an IRES element. The env* construct contains a frameshift in *env*. Restriction sites used for cloning are shown in italics.

Currently, XMRV infection and replication is quantified by measuring proviral DNA numbers in infected cell cultures, or by determining virion associated genomic RNA copy numbers and reverse transcriptase activities in the supernatants of infected cells [24,25,36,37]. Alternatively, productive infection with release of infectious XMRV has also been analyzed by titrating progeny virus on reporter cell lines [26,38]. These techniques are relatively time consuming, do not allow a high throughput analysis, and are relatively expensive. For sensitive and convenient monitoring of XMRV infection and replication, we sought to generate replication-competent XMRV reporter viruses encoding an enhanced form of the green fluorescence protein (eGFP, named GFP hereafter) [39], or the secreted *Gaussia* *princeps* luciferase (GLUC) [40] via an internal ribosomal entry site downstream of the XMRV *env* frame (Figure 1). For this, we obtained the pCR2.1-TOPO XMRV-VP62 proviral clone [1] which lacks the U3 region in the 5`LTR, termed XMRV-Δ5U3 (Figure 1). Since restriction mapping of this plasmid revealed unexpected restriction sites in the vector backbone, we sub-cloned the XMRV-Δ5U3 proviral DNA into the pCR2.1TOPO vector. Next, a PCR amplified *IRES-GFP* cassette was inserted into the unique PmlI site 3' of the *env* ORF resulting in the generation of the plasmid XMRV-GFP-Δ5U3 (Figure 1). The lacking U3 region in the 5`LTR was reconstituted with the U3 region in the 3`LTR by a PCR-based approach (see material and methods for details) resulting in a XMRV proviral construct encoding the complete 5'LTR, *gag, pol* and *env*, followed by an *IRES-GFP* element and the 3' U3-R region (XMRV-GFP) (Figure 1).

The variant encoding the secretable luciferase (XMRV-GLUC) was generated by exchanging the *IRES-GFP* with an *IRES-GLUC* fragment. As controls, we also introduced a frameshift in env (XMRV-env*-GFP/GLUC) (Figure 1). The generated XMRV reporter virus DNAs were sequenced on both strands and digested with a panel of restriction enzymes confirming the presence of all indicated alterations (Figure S1). Notably, gene expression in all generated constructs is driven by the wt XMRV promoter in the 5`LTR and not by a strong CMV promoter as described for other XMRV variants [41,42].

### Production of XMRV reporter viruses

**Figure 2:**
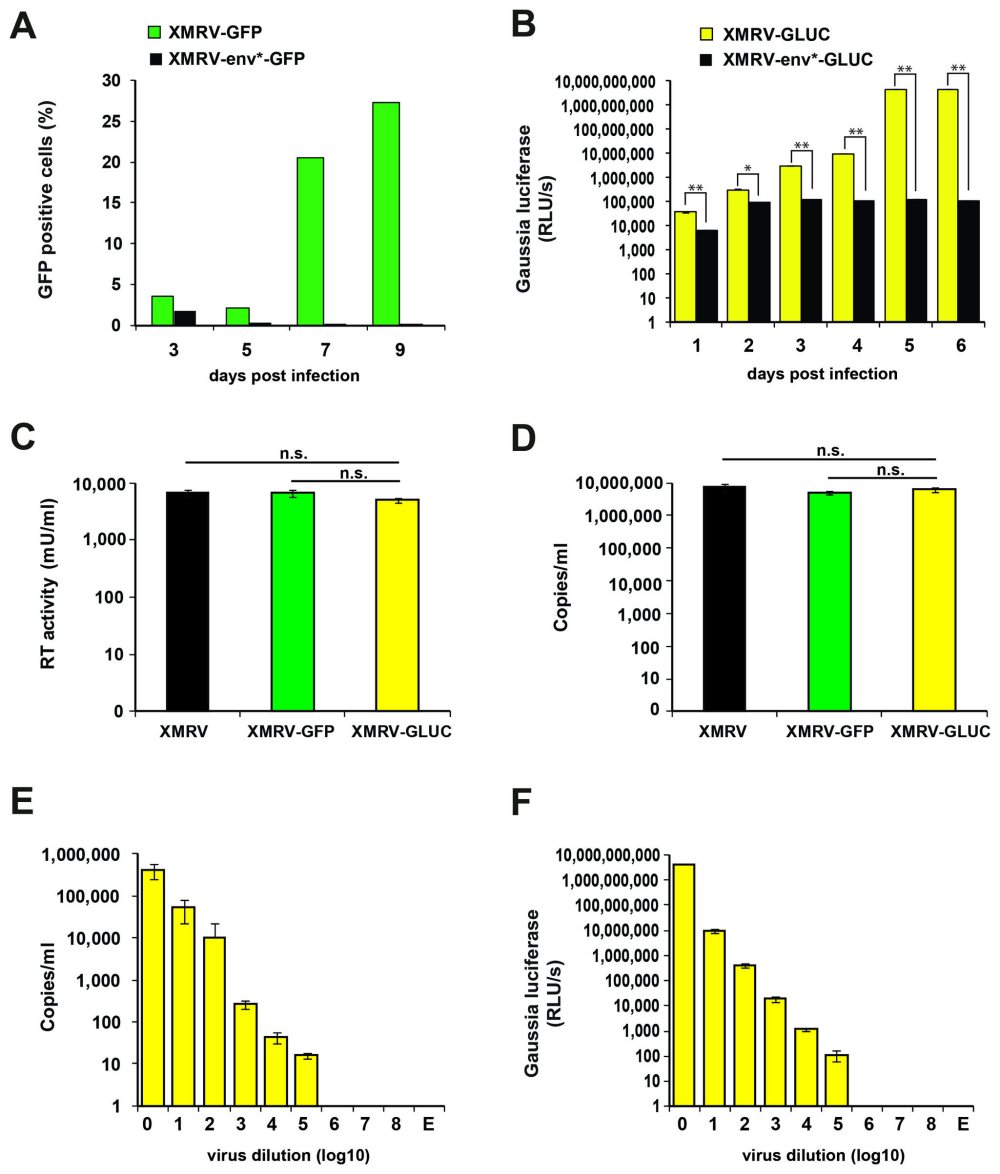
XMRV reporter gene expression and virion production. A) Flow cytometry analysis of LNCaP cells transfected with *env* intact or *env* defect XMRV-GFP proviral DNA. B) GLUC activities in the supernatants of LNCaP cells transfected with *env* intact or *env* defect XMRV-GLUC constructs. C) Reverse transcriptase activities and D) Genomic RNA copy numbers (D) in supernatants of LNCaP cells transfected with XMRV wt, XMRV-GFP and XMRV-GLUC proviral DNA. E) qPCR and F) *Gaussia* luciferase activities of serial dilutions of an XMRV-GLUC virus stock. Values shown are average values derived from triplicate measurements ± standard deviation. Abbreviations and symbols: RLU/s (relative light units per second), n.s. (not significant), E (medium only), * (p<0.001), ** (p<0.00001).

To produce infectious XMRV reporter viruses and to study whether the cloned reporter genes are expressed, we transfected proviral reporter constructs using a Lipofectamine-based method into LNCaP cells. Flow cytometry analysis of pCR2.1_XMRV-GFP transfected cells showed that approximately 4% of the cells transfected with the *env*-intact plasmids and 2.5% of the cells transfected with the *env*-defect proviruses were GFP positive after 3 days (Figure 2A). Control transfections performed with a GFP expression plasmid yielded only moderately increased numbers of GFP positive cells (data not shown) suggesting that the low percentage of GFP positive cells was not due to reduced GFP expression of the provirus but a generally low transfection efficiency. In contrast to samples transfected with the *env*-defect construct in which GFP expressing cells disappeared over time, the number of fluorescent cells transfected with *env-*intact XMRV-GFP increased over time suggesting viral spread in cell culture (Figure 2A).

Transfection of LNCaP cells with *env*-intact XMRV-GLUC resulted in a time- and DNA concentration dependent increase of *Gaussia* -luciferase activities in cellular supernatants (Figure 2B). At late time points, luciferase activities reached the upper detection limit of the luminometer (> 4.6 x 10^9^ RLU/s) demonstrating the high sensitivity of this assay system. As expected, no increase of GLUC activities over time was observed after transfection with the *env*-defect XMRV-GLUC provirus (Figure 2B).

We next analyzed whether cells transfected with proviral XMRV-DNA released viral particles into the supernatant. For this, we determined virion associated reverse transcriptase (RT)-activities and RNA copy numbers derived from supernatants of XMRV-wt, XMRV-GFP and XMRV-GLUC transfected LNCaP cells. We detected similar RT-activities (Figure 2C) and RNA copy numbers (Figure 2D) suggesting that the transfected cells produce virions and that the increased genomic size of the XMRV reporter constructs compared to wt XMRV has no significant effect on virus yield. To compare the sensitivities of the luciferase with the quantitative PCR assay, serial dilutions of the XMRV-GLUC stock were analyzed. Both methodologies allowed detection of a signal in the 10^5^ but not the 10^6^-fold dilution (Figure 2E and F). Thus, the detection of GLUC activities in cellular supernatants by a relatively fast and inexpensive assay achieves similar results as to those which were obtained by quantitative PCR determining RNA copy numbers. All together, these results demonstrate that the generated proviral XMRV constructs express the reporter gene, spread in provirally transfected LNCaP cells and produce progeny virus.

### XMRV reporter viruses are infectious and replication competent

**Figure 3:**
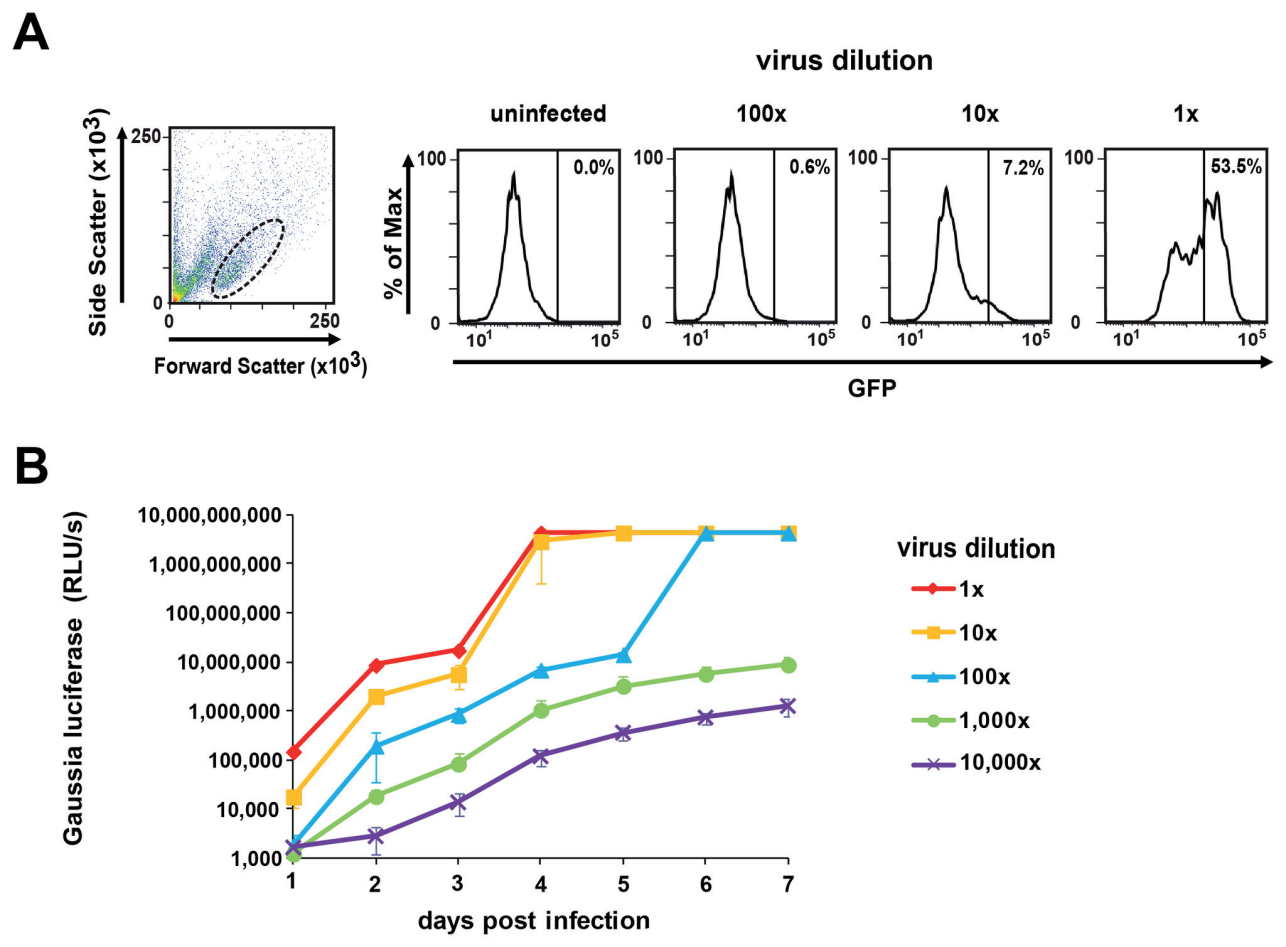
XMRV reporter viruses are infectious. A, B) Serial dilutions of XMRV-GFP (A) or XMRV-GLUC (B) were used to inoculate Raji cells. Infection rates were determined by measuring GFP expression 3 days later (A) or by quantifying GLUC activities in cellular supernatants obtained at indicated time points (B). RLU/s (relative light units per second).

In the next set of experiments we determined whether virions produced from transfected LNCaP cells are infectious and replication-competent. We inoculated LNCaP cells with cell-free XMRV-GFP, and observed a dose-dependent increase of fluorescent cells, with a maximum of 53.5% GFP positive cells after infection with undiluted virus (Figure 3A). Inoculation with the *env*-defect construct did not result in detectable GFP expression (data not shown). To address whether virions released from infected LNCaP cells are capable of initiating a new round of infection, we performed passaging experiments in highly permissive Raji cells (see below). Even after the 7th passage, we detected a high percentage of GFP positive cells by UV microscopy (data not shown) demonstrating stable reporter gene expression over multiple rounds of viral replication. Thus, the XMRV-GFP construct is infectious and replication competent, and allows for the detection of XMRV on a single cell level by flow cytometry.

To analyze the functionality of the *Gaussia* luciferase virus, LNCaP cells seeded in microtiter plates were infected with dilutions of cell free XMRV-GLUC stock. As expected, GLUC activities in supernatants correlated with the amount of input virus, and increased over time (Figure 3B). After infection with high viral doses, reporter gene activities reached the maximum detection limit of the luminescence detector, and even a 10,000-fold dilution of the virus still resulted in detectable enzyme activities starting from day two post infection (Figure 3B). These results show that inoculation of LNCaP cells with XMRV-GLUC is a highly sensitive and convenient method to quantify XMRV infection. Accordingly, XMRV-GLUC could also be passaged in Raji cells demonstrating that the reporter virus is replication competent and does not lose its reporter cassette over at least five rounds of passaging (data not shown), similar to the GFP variant.

The XMRV-GLUC reporter virus provides a major advantage over the wt virus as it permits a rapid and accurate high throughput analysis e.g. of antiviral drugs. To test this, we determined the anti-XMRV activity of Azidothymidine (AZT), a reverse transcriptase inhibitor, in a time-course experiment. AZT resulted in a dose-dependent decrease of reporter activities (Figure S2). The broad linear range of the GLUC detection assay also permitted to conveniently calculate the IC_50_ value of AZT which was e.g. 5 ± 1.1 nM at day 4 post infection (Figure S2). To our best knowledge, the generated XMRV-GLUC and XMRV-GFP constructs represent the first replication-competent XMRV reporter viruses to date and should greatly facilitate future research on gammaretroviruses.

### XMRV infects a variety of immortalized human cell lines

We next investigated XMRV susceptibility of human cells and inoculated various cell lines with XMRV-GFP. We found that all tested cell lines supported XMRV infection but reporter gene expression varied greatly (Table 1). In agreement with previous reports, XMRV infected DU145 prostate cancer cells less efficiently than LNCaP cells [21]. For T cell lines, we observed infection rates that ranged from 1.2% (PM1 cells) to 11.9% (Jurkats) [20], which is also in agreement with published data. Infection of the glioblastoma cell line U87 and U373 resulted in relatively low infection rates (3-4% GFP positive cells) which his is in line with a recent article reporting a reduced XMRV permissiveness of astrocytes [43]. Similar results were obtained using the XMRV-GLUC reporter virus (Figure S3). As previously published, mouse NIH3T3 fibroblasts were largely refractory to XMRV (Figure S3) [44] which is in accordance with data showing that the XPR1 receptor of NIH 3T3 cells does not permit XMRV infection [45].

**Table 1:** XMRV infection of human cell lines.

**Name**	**Cell type**	**Infection (% GFP**)
LNCaP	Prostate cancer cell line	53.6
DU145	Prostate cancer cell line	1.9
HEK293T	Human embryonic kidney cell line	12.4
HeLa	Cervical cancer cell line	0.3
U87	Glioblastoma cell line	3.6
U373	Glioblastoma cell line	3.3
Jurkat	T cell line	11.9
JTag20	T cell line	8.8
CEM	T cell line	2.9
A3.01	T cell line	1.8
SupT1	T cell line	1.8
PM1	T cell line	1.2
BJAB	B cell line	95.4
Raji	B cell line	72.3

Indicated cell lines were inoculated with XMRV-GFP and fluorescence protein expression was measured 3 days after inoculation by flow cytometry.

**Figure 4:**
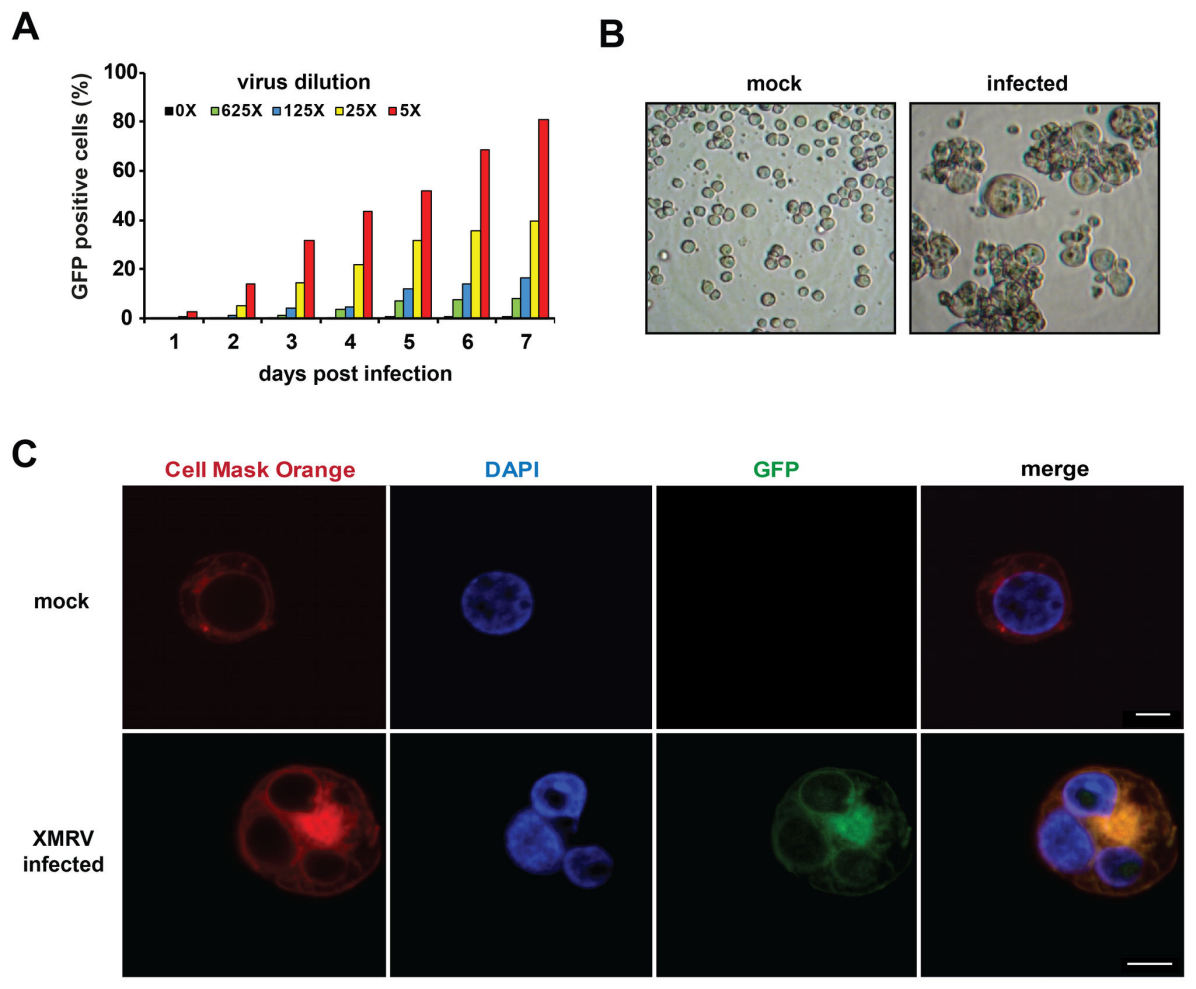
XMRV replicates efficiently in Raji B cells and causes a cytopathic effect. A) Raji cells were infected with the indicated dilutions of XMRV-GFP and expression of the fluorescence protein was analyzed by flow cytometry at indicated time points. B, C) Formation of multinucleated syncytia in XMRV-GFP infected Raji cells as shown by light microscopy (B) and confocal microscopy (C). Scale bar 5 µm.

Compared to most other cell types, the B cell lines BJAB and Raji were highly permissive for XMRV (Table 1). For example, flow cytometry analysis of infected Raji cultures infected with serial dilutions of XMRV-GFP revealed a time and viral-dose dependent increase in the number of GFP positive cells (Figure 4A, Figure S4) suggesting effective viral spread. Surprisingly, light microscopy analysis of XMRV infected Raji cells revealed the presence of syncytia (Figure 4B), a phenotype that we never observed for XMRV in other cell lines, and which to our best knowledge has not been reported for XMRV before. Confocal microscopy of these cultures confirmed the presence of GFP-positive syncytia which contained more than one nucleus (Figure 4C). It has recently been shown that expression of XMRV Env variants carrying truncations in the C-terminal cytoplasmic tail promote syncytia-formation in 293 cells [46]. It is also well established that some ecotropic gamma retroviruses are capable of forming syncytia, and that XMRV-related polytropic viruses are cytopathic in some cells, most likely because they fail to establish superinfection immunity [47-50]. However, although the mechanism(s) underlying the formation of syncytia in Raji cells are currently unclear, our results confirm and expand previous findings demonstrating that XMRV is capable of infecting a variety of immortalized human cells. The observed differences in XMRV susceptibility between cells are likely mediated by differential activities of cellular restriction factors, but also host gene polymorphisms might contribute since all tested cell lines were observed from different human donors.

### XMRV does not spread in primary human cells

**Figure 5:**
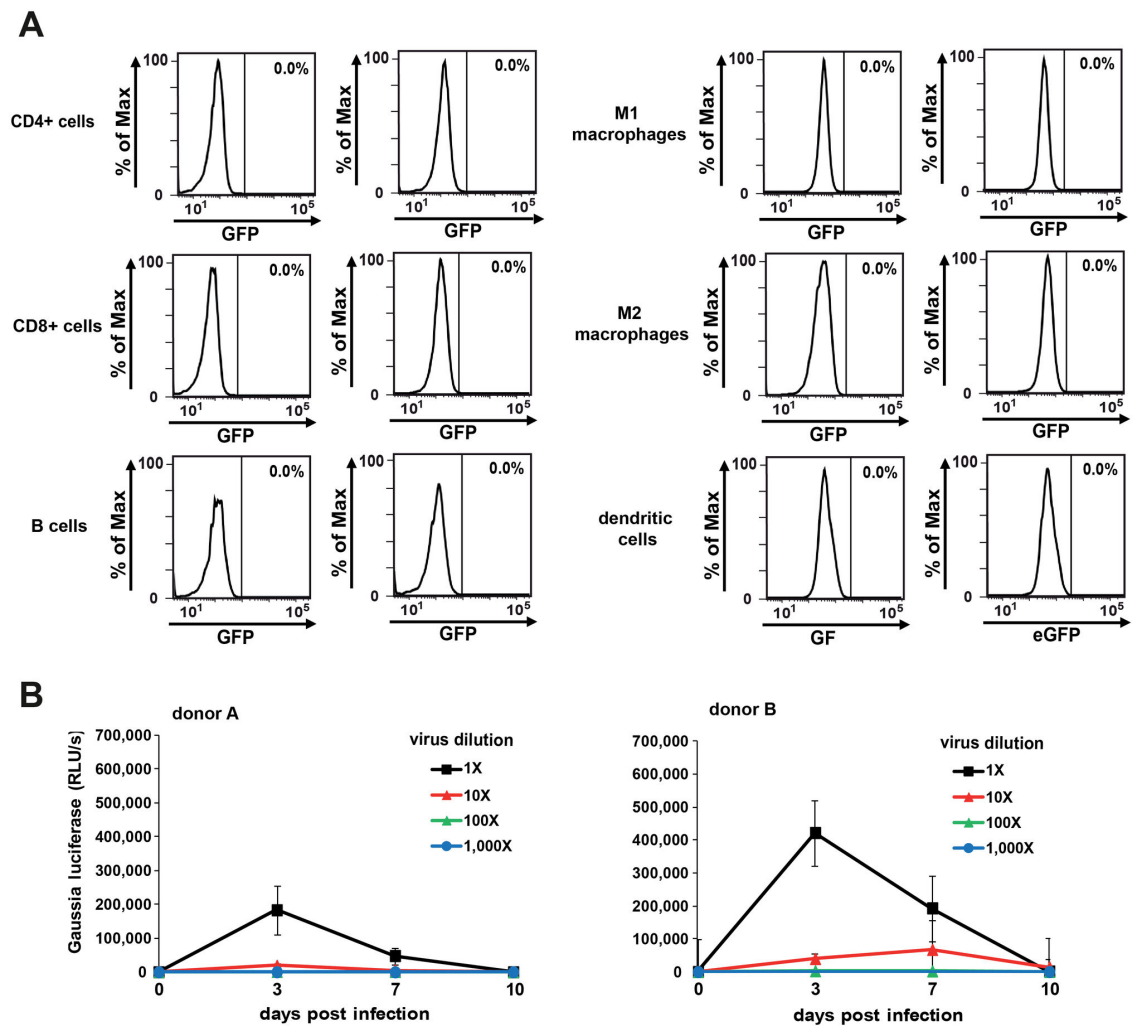
XMRV infection is severely restricted in primary human cells. A) Flow cytometry analysis of primary human cell types inoculated with XMRV-GFP. Cells were cultured as described in the material and methods part and inoculated with supernatant of XMRV-GFP. Flow cytometry was performed 3 days later. B) PHA/IL-2 stimulated PBMC from two donors were infected with serial dilutions of XMRV-GLUC and enzyme activities were quantified in supernatants obtained at indicated time points. Values represent average reporter activities derived from triple infections; RLU/s (relative light units per second).

To be a genuine human pathogen, XMRV must be capable of infecting primary human cells. Since XMRV was described to be found in and isolated from peripheral blood mononuclear cells (PBMC) [2], we first sought to analyze which PBMC subsets are susceptible to XMRV. For this, we inoculated primary CD4 and CD8+ T cells, B cells, macrophages of the M1 and M2 type, and dendritic cells with an undiluted XMRV-GFP stock which has previously been shown to be highly infectious in Raji cells (Figure 3A). Flow cytometry performed at various days post inoculation never resulted in significant quantities of GFP positive cells (example shown in Figure 5A), and similar results were obtained using cells from different donors. Thus, XMRV gene expression seems to be severely restricted in the analyzed primary cell subsets.

We next studied XMRV infection and replication in whole PBMC cultures. For this, we inoculated non-stimulated freshly obtained PBMC with serial dilutions of an XMRV-GLUC stock which readily infects and replicates in Raji cells (Figure 3B). In contrast to the immortalized B cells in which luciferase activities reached the upper detection limit of the luminometer (Figure 3B), we could not detect any increase in luciferase activities over background in supernatants of XMRV exposed non-stimulated PBMC of different donors (data not shown). These results demonstrate that XMRV gene expression and hence replication is severely impaired in nonstimulated PBMC. Next, we analyzed if activated PBMC allow XMRV infection and replication. As expected from previous reports [24–26], inoculation of PHA/IL-2 stimulated PBMC with serial dilutions of highly infectious XMRV-GLUC stock resulted in detectable reporter gene activities derived three days post infection (Figure 5B). However, enzyme activities decreased at later time points, and inoculation with higher viral doses which were infectious in Raji cells (Figure 3B) did not cause detectable GLUC activities in PBMC supernatants (Figure 5B). These data show that XMRV is principally capable of infecting activated PBMC which results in viral gene expression, but compared to immortalized cells, infection or gene expression is restricted.

In the next set of experiments we tested whether XMRV infected stimulated PBMC release infectious progeny virus. Therefore, we inoculated highly susceptible Raji cells (Figures 3 and 4) with GLUC-positive supernatants derived at day 3 and 7 from activated PBMC (Figure 5B). We never detected any luciferase expression or signs of an XMRV induced cytopathic effect, even after two weeks of continuous cell culture (data not shown). Thus, although XMRV is capable of infecting stimulated PBMCs it does not establish a productive and spreading infection in those cells that have previously been reported to be infected with XMRV *in vivo* [2].

## Conclusions

We here report the generation of first replication-competent XMRV reporter viruses that express GFP or a secretable luciferase from a bicistronic env-IRES-*GFP*/*GLUC* cassette. Transfection of proviral DNAs into LNCaP cells resulted in readily detectable reporter gene expression as well as production of progeny XMRV particles. Inoculation of known XMRV permissive cells such as LNCaP or Raji [2,21] showed that the generated reporter viruses were infectious and expressed the encoded reporter genes, allowing for a fast and convenient quantification by luminescence or flow cytometry based methods. In addition, XMRV-GFP and XMRV-GLUC could be passaged in Raji cells over multiple rounds of infection without losing their reporter gene cassettes. XMRV tropism and replication has previously been analyzed by applying various methods like nested PCR [51], RT-PCR [21], Western blotting [2], fluorescence in situ hybridization (FISH) [1], immunohistochemistry (IHC) [1], reverse transcriptase assay [21], or utilization of XMRV-pseudotyped virions [52]. These techniques all have specific advantages but also limitations such as small sample size, use of replication deficient viruses, low quantification accuracies, or high costs.

The use of the newly developed XMRV-GFP reporter permits the analysis of a replication competent γ-retrovirus by fluorescence microscopy and/or flow cytometry opening a plethora of applications. These include e.g. single cell analysis to quantify the effect of viral gene expression on cell surface markers, the identification of infected cellular subsets, or the visualization of spatial and temporal patterns of infection, *in vitro* and *in vivo* [53–56]. The GLUC-expressing construct will certainly facilitate future studies on XMRV/MLV biology because the luciferase is secreted into the supernatant of infected cells where it can be detected in a rapid, cheap, accurate and sensitive assay. Thus, viral replication can be monitored over time without the need to lyse the cells. In addition, *Gaussia* luciferase produces the highest number of photons per mol making it orders of magnitude more sensitive than renilla or firefly luciferase [57,58]. We also found that quantification of GLUC in virus stocks is as sensitive as the detection of viral RNA copies by qPCR (Figure 2) - while the detection of the luciferase is significantly less expensive and less time consuming. Previous studies demonstrated that GLUC can be detected in blood suggesting that XMRV-GLUC reporter viruses might present a sensitive and convenient tool to indirectly monitor viral loads *in vivo* [40,59].

We initially constructed these viruses to study XMRV tropism and manipulation of primary human cells. We found that XMRV enters and replicates efficiently in a variety of immortalized human cell lines, confirming previous results [20,21,43,44]. Both reporter viruses could be passaged in Raji cells over multiple rounds of infection, and unexpectedly also resulted in the formation of syncytia in these cells. However, inoculation of primary PBMC bulk cultures or isolated cellular subsets with XMRV stocks that were highly infectious in Raji cells never resulted in detectable reporter gene expression. Although these results do not exclude that XMRV successfully entered non-stimulated PBMCs, they suggest that viral gene expression is severely impaired. In contrast, when PHA/IL-2 activated PBMC were inoculated, GLUC activities could be readily detected, showing that XMRV is capable of infecting the cells resulting in proviral DNA integration and viral gene expression. However, compared to Raji cell infections, GLUC activities in activated PBMC cultures were significantly lower and decreased over time, indicating abortive infection. Indeed, inoculation of Raji cells with GLUC-containing supernatants derived from activated PBMCs never resulted in new rounds of infection. Thus, PBMCs do not release infectious progeny virus confirming and expanding results of previous studies which used quantitative RT-PCR [25], nested PCR [24] or DERSE-iGFP reporter cells [26] to study XMRV infection and replication in PBMC. Impairment of XMRV replication in PBMCs has been attributed to the restriction factors APOBEC3G and 3F [25,28]. Taken together, XMRV can efficiently replicate in some human cell lines but not in primary PBMC supporting recent evidence that XMRV is not a genuine human pathogen.

## Supporting Information

Figure S1 **Digestion with a panel of restriction enzymes.** Agarose gel electrophoresis of restriction digestions of the proviral XMRV plasmids with EcoRI, HindIII, NheI, SacI, SalI and XmaI. 1 µg DNA was digested for 1 hour with 1 µl of the indicated enzymes. Loading dye was added to the samples before running on a 0.8% agarose gel. A 1 kb DNA ladder was loaded on the gel for visualization of the band sizes.
(TIF)

Figure S2 **Antiviral activity of AZT against XMRV.** Raji cells containing the indicated concentrations of the reverse transcriptase inhibitor AZT were infected with GLUC encoding XMRV. 2, 4 and 6 days post infection, supernatants were taken, 100-fold diluted, and GLUC activities were determined in cellular supernatants. The left panel shows raw data, the right panel depicts the % inhibition rates obtained at 4 days post infection. RLU/s, relative light units per second.
(TIF)

Figure S3 **XMRV-GLUC infection of human cell lines and mouse NIH3T3 cells.** Different cell lines from human (Raji, LNCaP, HEK293T, Jurkat, U373 and HeLa) and mouse (NIH3T3) origin were infected with XMRV-GLUC reporter virus, harvested from transient transfected LNCaP cells, in 24 wells plate in 500 µl of media. Next day inocula were removed and cells were washed once with PBS and fresh media were added to the cells. Every day 50 µl of supernatants were harvested and same amount of fresh media were added. *Gaussia* activities were analyzed using 100-fold dilutions of the supernatants. RLU/s = relative light units per second.
(TIF)

Figure S4 **XMRV-GFP infection of Raji cells.** Raji cells were infected with XMRV-GFP virus (in 5-fold dilutions) in RPMI-10 media and analyzed at indicated days post infection by flow cytometry.
(TIF)
