# Supplementary figures and images for: Utilization of Replication-Competent XMRV Reporter-Viruses Reveals Severe Viral Restriction in Primary Human Cells
Source: PLoS One. 2013 Sep 13;8(9):e74427. doi: 10.1371/journal.pone.0074427 (PMC3772927; doi:10.1371/journal.pone.0074427)

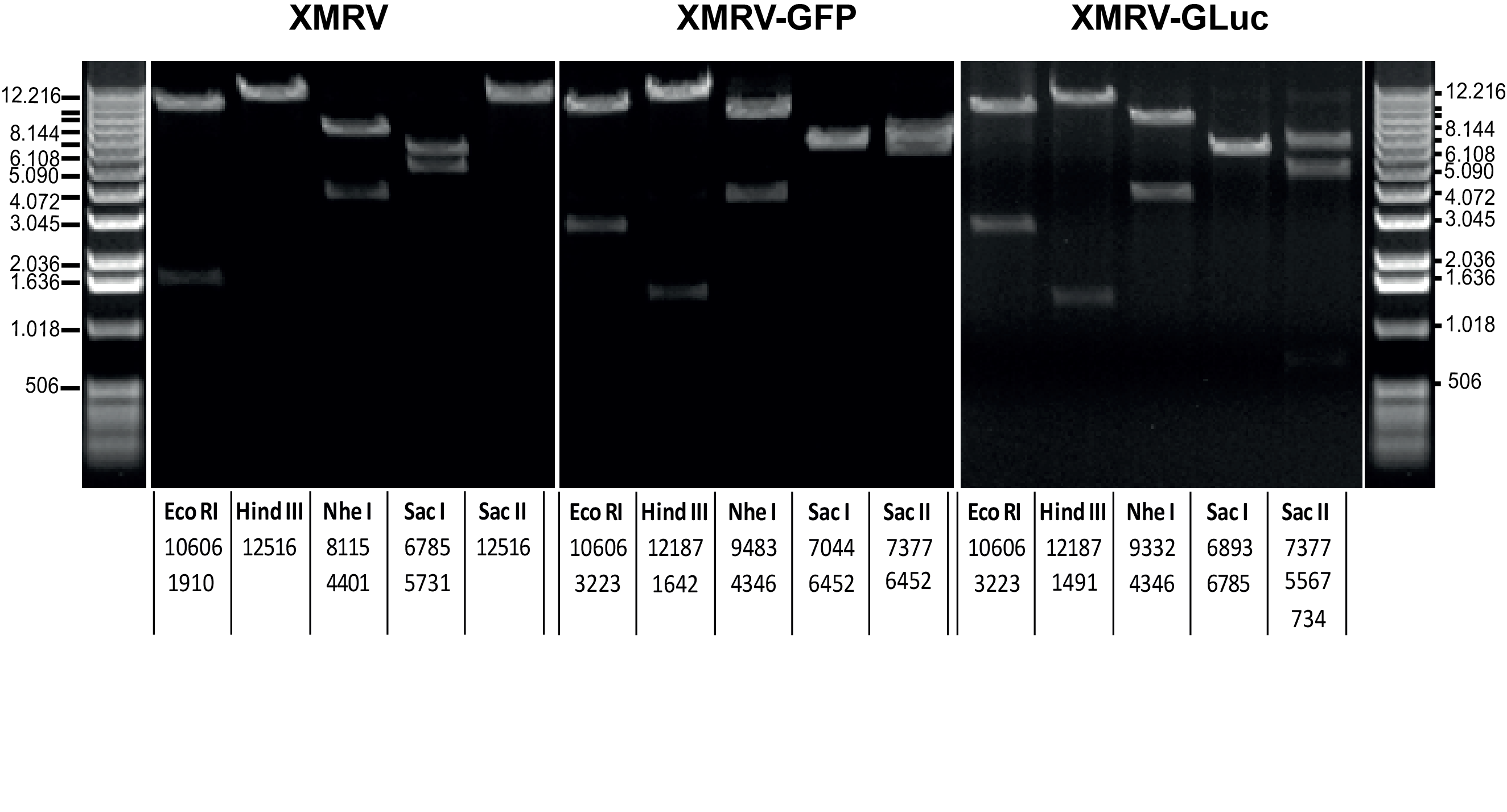

Supplement: Figure S1 — Digestion with a panel of restriction enzymes. Agarose gel electrophoresis of restriction digestions of the proviral XMRV plasmids with EcoRI, HindIII, NheI, SacI, SalI and XmaI. 1 µg DNA was digested for 1 hour with 1 µl of the indicated enzymes. Loading dye was added to the samples before running on a 0.8% agarose gel. A 1 kb DNA ladder was loaded on the gel for visualization of the band sizes. (TIF) [file pone.0074427.s001.tif]

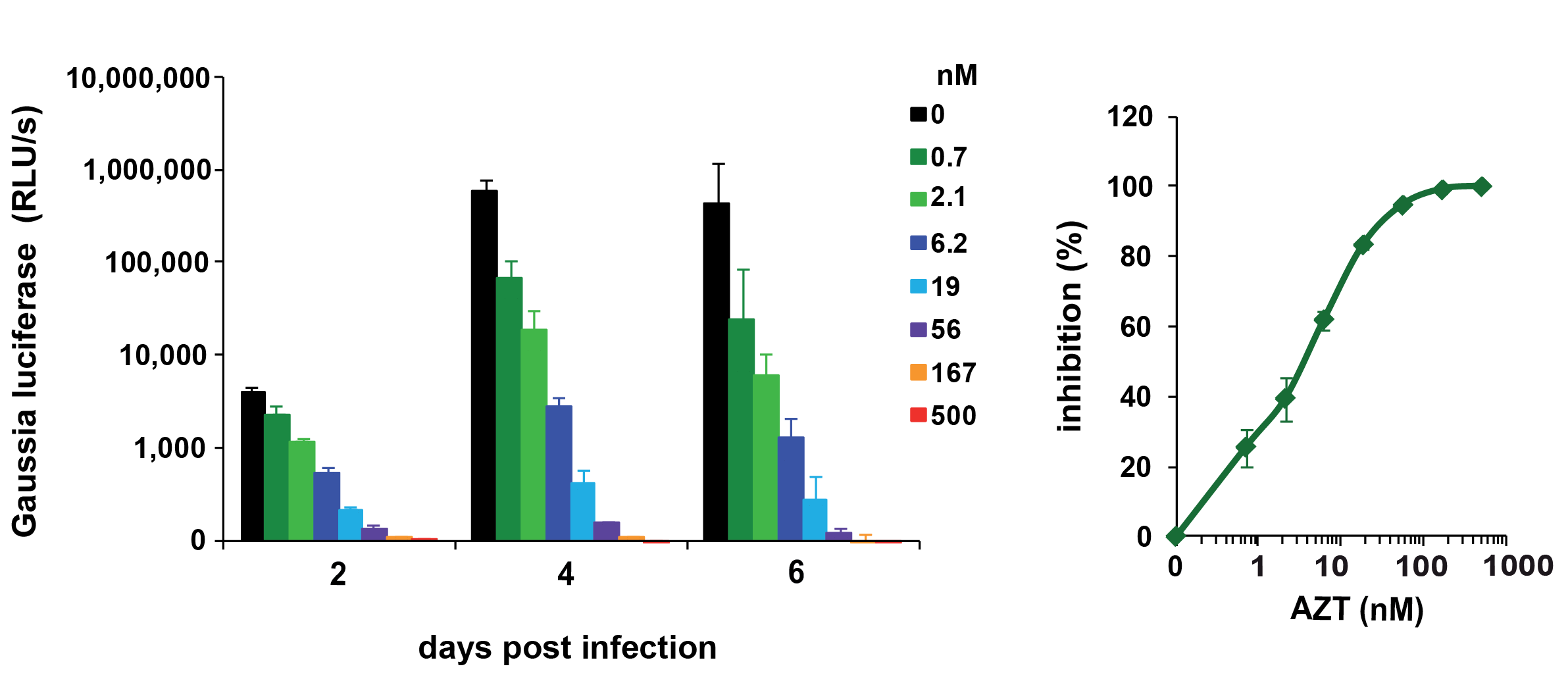

Supplement: Figure S2 — Antiviral activity of AZT against XMRV. Raji cells containing the indicated concentrations of the reverse transcriptase inhibitor AZT were infected with GLUC encoding XMRV. 2, 4 and 6 days post infection, supernatants were taken, 100-fold diluted, and GLUC activities were determined in cellular supernatants. The left panel shows raw data, the right panel depicts the % inhibition rates obtained at 4 days post infection. RLU/s, relative light units per second. (TIF) [file pone.0074427.s002.tif]

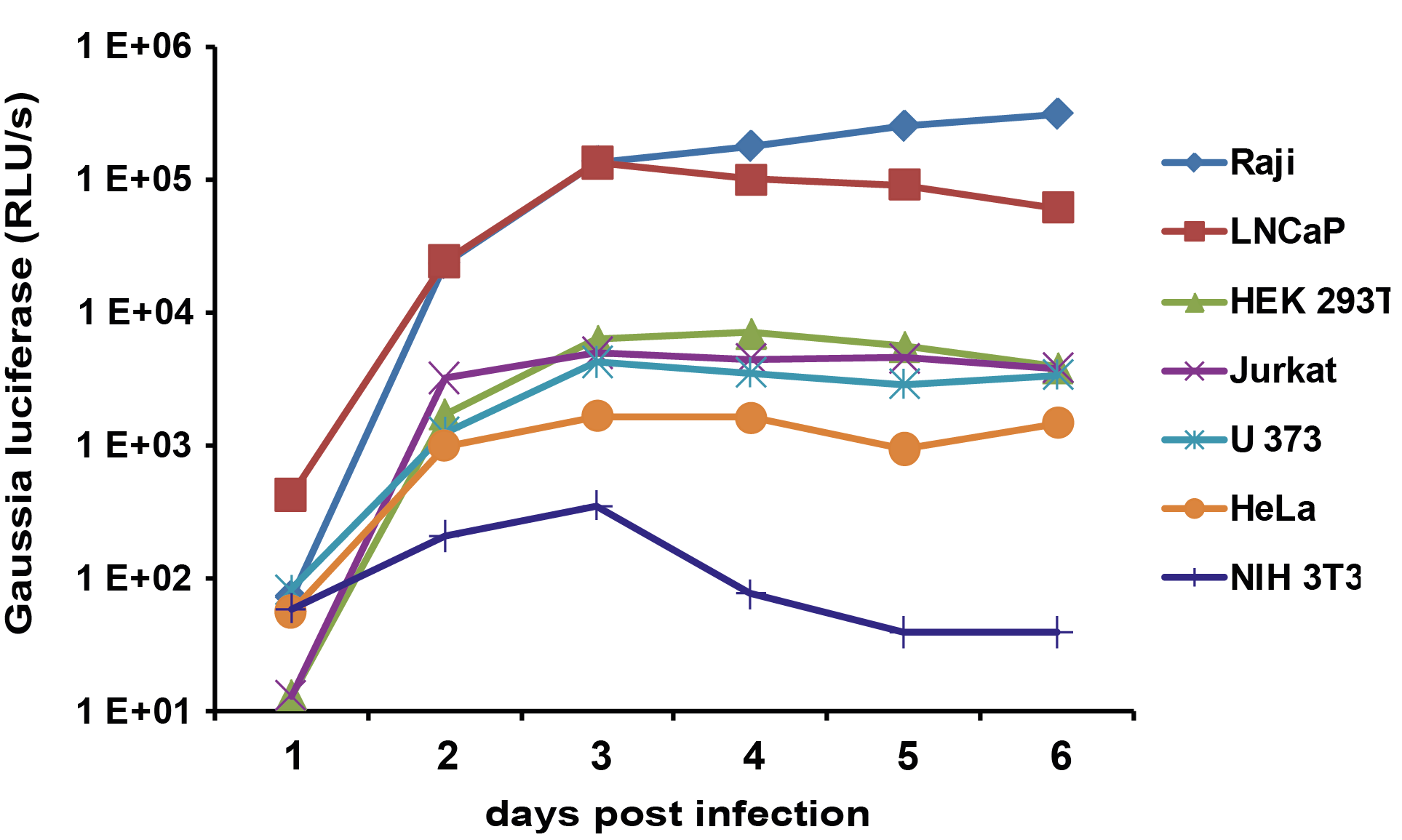

Supplement: Figure S3 — XMRV-GLUC infection of human cell lines and mouse NIH3T3 cells. Different cell lines from human (Raji, LNCaP, HEK293T, Jurkat, U373 and HeLa) and mouse (NIH3T3) origin were infected with XMRV-GLUC reporter virus, harvested from transient transfected LNCaP cells, in 24 wells plate in 500 µl of media. Next day inocula were removed and cells were washed once with PBS and fresh media were added to the cells. Every day 50 µl of supernatants were harvested and same amount of fresh media were added. Gaussia activities were analyzed using 100-fold dilutions of the supernatants. RLU/s = relative light units per second. (TIF) [file pone.0074427.s003.tif]

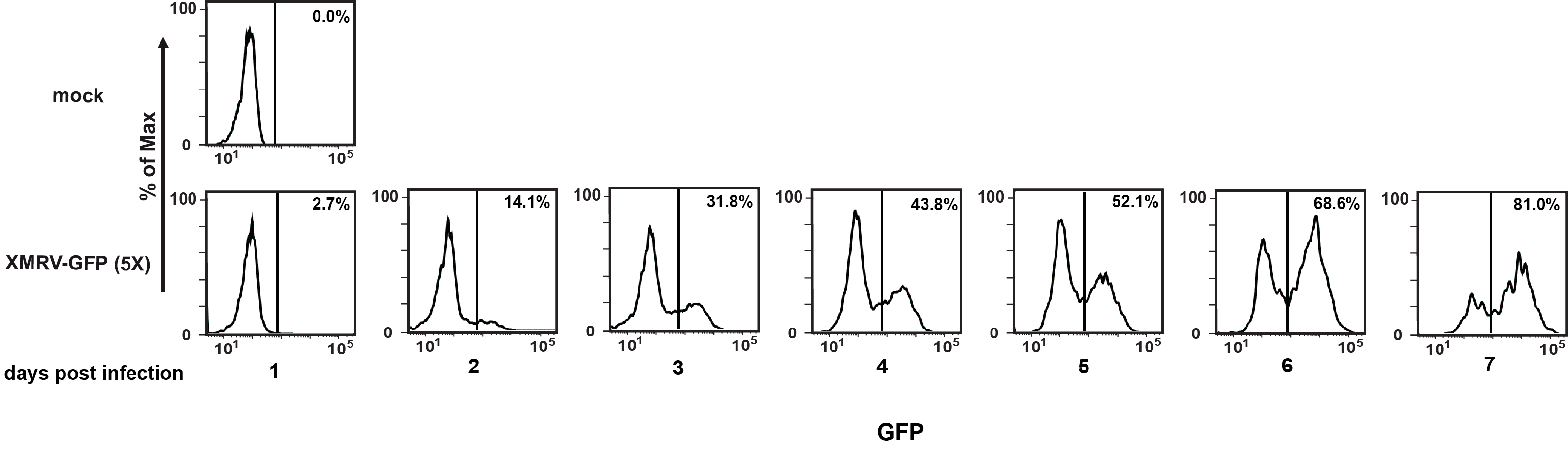

Supplement: Figure S4 — XMRV-GFP infection of Raji cells. Raji cells were infected with XMRV-GFP virus (in 5-fold dilutions) in RPMI-10 media and analyzed at indicated days post infection by flow cytometry. (TIF) [file pone.0074427.s004.tif]
